# The Nature Support Model for Dementia: a conceptual idea for green nursing home environments designed to support well-being throughout the last stages of dementia

**DOI:** 10.3389/fpsyg.2025.1567619

**Published:** 2025-06-13

**Authors:** Victoria Linn Lygum, Nanet Mathiasen, Anne Kathrine Frandsen

**Affiliations:** Department of the Built Environment, The Faculty of Engineering and Science, Aalborg University, Copenhagen, Denmark

**Keywords:** dementia, nursing home, health design, landscape architecture, nature, green environment

## Abstract

Dementia is one of the world’s greatest public health challenges. An increasing number of studies point to contact with nature in the form of green views and stays in green environments as a non-pharmacological way of supporting the well-being of persons with dementia. This article introduces the Nature Support Model for Dementia, a conceptual idea that can be used to design green environments at nursing homes for people with dementia intended to support the user’s health and well-being. The model is evidence-based and reposes on studies on user specific design of green environments. Furthermore, it builds on existing conceptual models for the design of supportive green environments. It caters to nursing home residents in the final stages of dementia decline. It considers their increasing needs for assistance and meets the needs for safety and security. The model was developed in connection with an evidence-based design process resulting in an architectural proposal for a nursing home. A presentation of this proposal illustrates how the model can be used in practice to facilitate architectural programming and the design of green nursing home environments intended to be supportive.

## 1 Introduction

### 1.1 Dementia–a public health challenge

Dementia is one of the world’s greatest public health challenges. In 2019, 55.2 million people had dementia worldwide and by 2050, this number is estimated to rise to 139 million (World Health Organization, 2021). Dementia is an umbrella term for several dementia diseases, of which Alzheimer’s syndrome is the most common (Nationalt Videnscenter for Demens, 2021). Dementia is characterized by a gradual decrease in the diagnosed person’s condition, getting progressively worse until death. There is no cure for most of dementia diseases, only medication that can postpone and relieve symptoms. How the disease manifests and its duration varies from person to person, but overall, cognitive skills deteriorate which can ultimately result in the loss of basic skills such as the ability to judge one’s own needs or recognize and swallow food (Nationalt Videnscenter for Demens, 2021, 2023). Research on approaches to prevention, treatment, care and cure for dementia is much needed (Prince et al., 2015). What is often required is a supportive physical environment, where treatment and care are available in a manner that is tailored for people with dementia. This is easier said than done. In Denmark, only the weakest of elderly people are offered accommodation at a public nursing home and on average those who qualify only get to live at the nursing home for two years and six months (ÆldreSagen, 2023). In 2021, 51,700 people over the age of 75 lived in a Danish nursing home (Danmarks Statistik, 2021), of whom 75 to 80% had dementia (Alzheimerforeningen, 2022). In addition to catering to residents with poor health, nursing homes are also under pressure from other factors, including high staff turnover, a lack of professional training (Sparre et al., 2023), and inappropriate physical settings (Kähler, 2014).

### 1.2 The stages of dementia decline

The Global Deterioration Scale (Reisberg et al., 1982) is a tool used to determine the stages of cognitive function among people diagnosed with dementia and describes seven stages from cognitively normal to very severe dementia. Stage one (no cognitive decline) to stage four (moderate cognitive decline) covers mild dementia with symptoms such as subjective work difficulties and a decreased ability to handle complex assignments. Stage five (moderately severe cognitive decline) is the point where a person can no longer live without assistance. People at this stage have difficulty making decisions and organizing and performing everyday tasks (Reisberg, 2007). They require assistance finding proper clothing to wear for the day, for example. Stage six (severe cognitive decline) is characterized by a need for assistance in most daily activities such as bathing and visiting the toilet. Urinary and fecal incontinence is also part of this stage. On the last and seventh stage (very severe cognitive decline) the ability to speak and walk is limited. At the furthest extents of the condition’s progression, a person’s ability to sit up and hold up their head independently is also lost (Reisberg, 2007). Despite these defined stages, the symptoms of dementia are expressed in very different ways depending on the person and context. Related to the physical environment symptoms of dementia can result in problems understanding surroundings, situations and finding way (Marshall, 2019).

### 1.3 Inappropriate use of antipsychotics

In fear and helplessness, people in the last stages of dementia can externalize their frustration and aggression. Among other things, this is one reason for the use of antipsychotic medication that can sedate the elderly and enable the staff to maintain basic aspects of everyday life such as getting clothes on and off. However, the use of antipsychotics is inappropriate as the brains of people with dementia are more sensitive to the medicine than those of healthy people (Falsing, 2021) as it weakens the cognitive functions that are already weakened by dementia (Sundhedsstyrelsen, 2024). The use of antipsychotics is associated with overdoses and side effects in the form of passivity, lethargy, and falls. In the worst cases their use can lead to premature death (Falsing, 2021). From a human rights perspective, the use of antipsychotic medication raises several ethical concerns, which calls for alternative ways of managing and caring for people with dementia.

### 1.4 Nature contact—a non-pharmacological alternative

Several literature reviews evidence the health benefits of contact with nature in nursing homes, where the most frequently cited benefits are decreased agitation (Detweiler et al., 2012; Gonzalez and Kirkevold, 2014; Whear et al., 2014; Lu et al., 2020; Zhao et al., 2019; Motealleh et al., 2019; Ng et al., 2023; Murroni et al., 2021), improved well-being and affect (Gonzalez and Kirkevold, 2014; Zhao et al., 2019; Ng et al., 2023; Murroni et al., 2021), improvement in attention (Detweiler et al., 2012; Lu et al., 2020; Zhao et al., 2019; Uwajeh et al., 2019; Motealleh et al., 2019; Ng et al., 2023; Murroni et al., 2021), and decreased use of medication (Detweiler et al., 2012; Gonzalez and Kirkevold, 2014; Murroni et al., 2021). Connecting with nature in nature-based activities not only improve mental well-being of people with dementia but also of family carers following activity sessions (Evans et al., 2022). However, to benefit from contact with nature, it is crucial that this contact is adapted to this specific user group. People with late dementia depend on assistance from caregivers because their initiative and ability to influence what they do and what others do to them is lost (Cohen-Mansfield et al., 2009). At the same time they depend on clear settings and activities that are easy to understand and participate in Marshall (2019). This calls for a physical setting specifically designed for the needs of people with dementia.

## 2 Materials and methods

### 2.1 The development of a model through evidence-based design

The objective of this theoretical research originates in a real-life architectural project for a new nursing home in Tune, Denmark and a need to find a strategy for planning the outdoor environment for nature to benefit the future users. The project happened in collaboration between the Greve Municipality, Housing Association Sjælland, the architectural firm Vandkunsten Architects and a team of researchers from the Department of the Built Environment, Aalborg University, Copenhagen. The part of the project concerning the incorporation of nature at the nursing home was organised as an evidence-based design process. Evidence-based design is a systematic process that involves research to formulate initial goals for the design and which ends in an evaluation of the goals and a publication of the results (Center for Health Design, 2010; Cooper Marcus and Sachs, 2014; Hamilton and Watkins, 2009; Stigsdotter and Sidenius, 2020). The process can be divided into four steps; the first step involves gathering knowledge, the second is about programming, the third focuses on design and construction of facilities, and the fourth includes an evaluation of the design (Stigsdotter and Sidenius, 2020). In this project, the first, second and third step led to the development of the model that was used as a tool in programming and design. The process included a series of meetings consisting of information exchange and collaboration between the team of researchers and the architects and landscape architects from the architectural firm and it was during these meetings that the model evolved. It helped communicate a strategy for planning the outdoor environment as it is a conceptual idea that combines knowledge from existing research on nature as a health-promoting resource as well as studies that consider user specific design of green environments at nursing homes. Furthermore, the model builds on existing conceptual models for the design of supportive green environments in general. The following gives an overview of this research and these concepts.

### 2.2 User specific design

Several publications consider how transitions between inside and outside environments and outdoor environments should be designed to benefit the users at nursing homes. They propose design guidelines and are generally grounded in a synthesis of research findings, practical experience, research on dementia, and architectural practice. The following themes can be drawn from reviews of design guidelines (Charras et al., 2017; Uwajeh et al., 2019; Motealleh et al., 2019):

- Attractive environments that provoke curiosity, sensory stimulation, and positive distraction
- Accessibility, orientation, movement, and ergonomics
- Space for social and solitary activities
- Meaningful activities and reminiscence
- Shelter, shade, safety, and security
- Sustainable planting and maintenance

Furthermore, a review of literature linking biophilic design strategies with nursing homes for people with dementia (Peters and Verderber, 2022) points to more detailed and conceptual key attributes including visual connections to nature; non-visual connections to nature (sound, tactility, smell, taste); non-rhythmic sensory stimuli (rustling leaves, gurgling water, wind in the grass); changes in temperature and air flow; the presence of water; dynamic and diffused light; complexity and order; prospect-refuge; and mystery (Peters and Verderber, 2022). Designing for biodiversity conservation by assuring many different types of habitats is important for the processes that support all life on Earth in general (Gagné et al., 2015) but it can also be a way of creating possibilities for connections to nature. Here a focus on regional flora and fauna might generate identification or familiarity between the users and the natural environment. Moreover, Marshall’s work on nursing environments and dementia in general brings forward the importance of views to nature from the bed as well as the possibility to get beds outside (Marshall, 2019).

### 2.3 Existing conceptual models

In addition to the guidelines described above, the following two existing conceptual models for the design of therapeutic landscapes were relevant for the development of the model. Scope of meaning/scope of action theory (Grahn et al., 2010) is applied as a theoretical background and determinant for the design of leading Scandinavian evidence-based therapy gardens, including the Therapeutic Garden at Alnarp, Sweden and Nacadia Forest Garden in Hørsholm, Denmark. Both of these are university projects and are used for recovery from exhaustion syndrome (Grahn et al., 2010; Stigsdotter and Randrup, 2008). Scope of meaning/Scope of action Theory considers the needs of people in crisis and how these needs can be met with physical environments. The theory claims that they seek simpler relationships which they find with elements of natural landscapes such as stones, plants, and animals. As recovery progresses, people seek more complex relationships such as those to other people. The theory is supported by a concept of four phases of rehabilitation each with their scope of action and coherent needs regarding the physical environment which can be catered to in the design of different areas of the garden. At the outset of the rehabilitation the individual in crisis tends to have a directed inwards involvement and seek environments where there is little to no human contact. This phase is succeeded by emotional participation, active participation, and finally outgoing involvement including activities with other people. When recovered, the need for a non-human environment is at its lowest. This relation is conceptually illustrated in Grahn et al. (2010) by an isosceles triangle which is horizontally divided in the four phases of rehabilitation where the need a supportive non-human environment decreases and scope of action increases from bottom to top. The design of the Therapeutic Garden at Alnarp and the Nacadia Forest Garden is consistent with this process of rehabilitation offering garden rooms tailored for each phase. Another design feature in the Nacadia Forest Garden concerns garden areas of varying safety and security which gradually increase in their levels of openness, going from closed to open (Stigsdotter and Randrup, 2008). This concept of degrees of closedness and openness is illustrated by Stigsdotter and Randrup (2008) with three ellipses where a small ellipse is embedded in a middle-sized ellipse, which again is embedded in a big ellipse. The three ellipses represent a graduation in the environment going from closed, to semi open to open supporting a recovery process where the needs for safety and security diminishes.

## 3 Result

### 3.1 The Nature Support Model for Dementia

Combining the existing guidelines and concepts, described above, to the needs of people with dementia, the Nature Support Model for Dementia gives a strategy for the inclusion of supportive green environments at nursing homes and can be used as a management tool in the design process linking the stages of dementia to needs in the physical environment.

**Figure 1:**
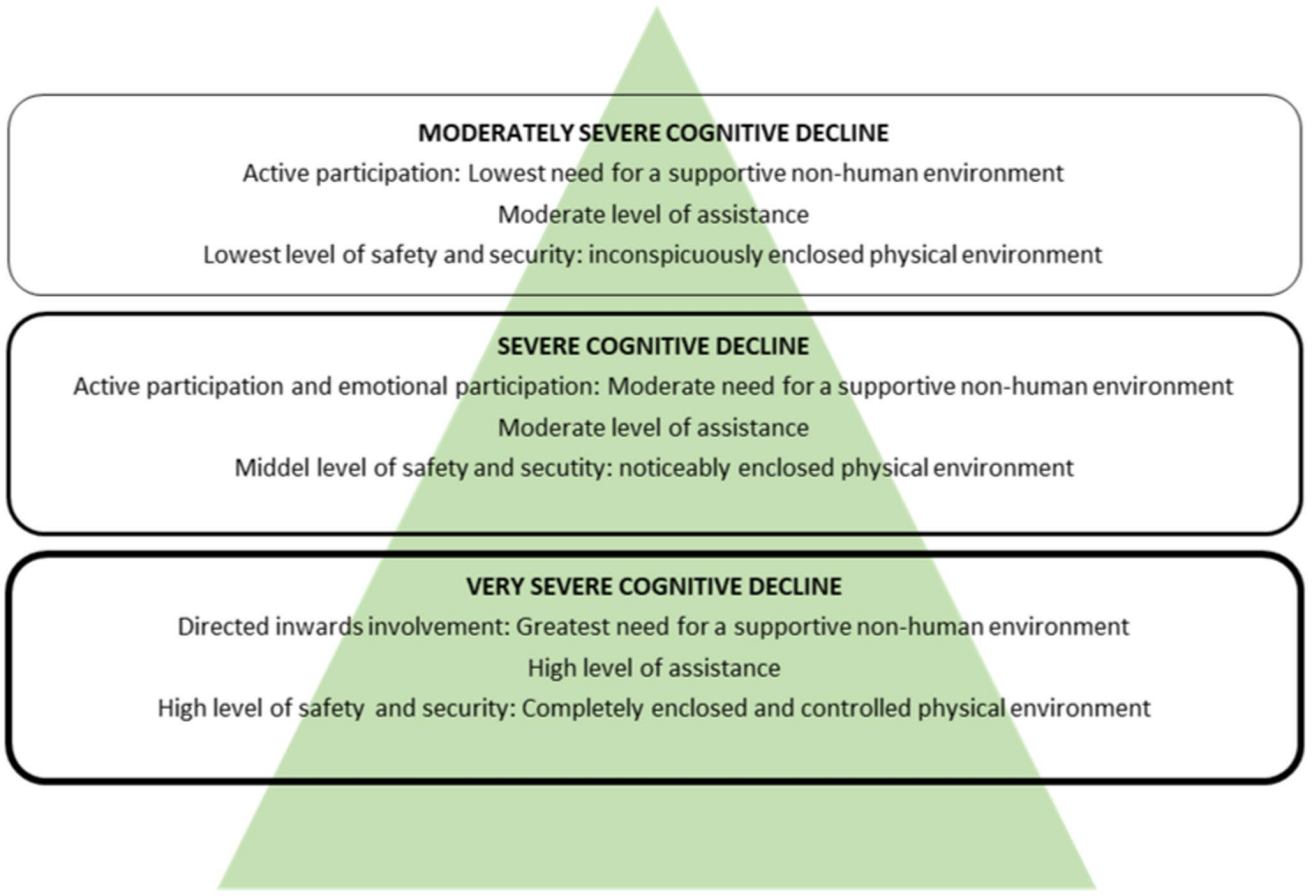
The Nature Support Model for Dementia.

It differs from previous models because it caters to the process of decline and not of recovery. As shown in Figure 1 (which illustrates the Nature Support Model for Dementia), the recovery process was reconceptualized into the process of decline to plan for supportive settings that foster well-being throughout the stages of dementia disease progression. By linking concepts from Scope of meaning/Scope of action Theory to the need for a safe and secure physical environment in the last stages of a dementia disease, three levels of needs can be described in relation to the setting of dementia care. The levels are divided according to people with moderately severe, severe, and very severe dementia, as these are the three stages most probable to be cared for in nursing homes.

The model illustrates the three last stages of decline from top to bottom with their increasing need for a supportive non-human environment and assistance as well as decreasing scope of action. The increasing need for a supportive non-human environment is illustrated by the shape of the triangle where the area of green is biggest at the bottom. Furthermore, the need for safety and security rises and is manifested, among other things, in increased levels of closedness in the physical environment going from inconspicuously enclosed to completely enclosed and controlled physical environments. This is illustrated by the increasing width of the line that encircles the textbox at each level.

The following is a description of the different types of green environment that correspond to the needs of people with dementia at the three last stages of decline, in accordance with the model.

Residents at stage five (moderately severe cognitive decline) have the lowest need for a supportive non-human environment, assistance, safety, and security. To challenge the residents positively, this requires a large, physical environment marked by inconspicuous boundaries giving gated access to eventual facilities in the surroundings of the nursing home. The green environment should provoke curiosity, stimulate the senses, and encourage reminiscence. At this stage the residents can participate actively in organized social and solitary activities. Focus should therefore be on meaningful activities, physical exercise, and socialization.

Residents at stage four (severe cognitive decline) have a moderate need for a supportive non-human environment as well as for safety and security. This calls for a mid-sized, physical environment that is noticeably enclosed from the surroundings. The need for a moderate level of assistance indicates that staff should be in the proximity of the garden along with facilities such as a kitchen and restrooms. In summary, easy orientation, and sheltered seating should give a sense of safety. Furthermore, the physical environment should have easy access to sensory stimulating greenery at standing and sitting height. At this stage the residents can still participate in staff organized social and solitary activities but may also have the need for just watching others being active in emotional participation.

Residents at stage seven (very severe cognitive decline) have the highest need for a supportive non-human environment, assistance, safety, and security. This calls for a small, closed, and controlled green environment; a prospect-refuge retreat that gives immediate contact to nature via views and a soothing atmosphere where sensory stimuli are adjustable to the individual. Residents at this stage can have a hard time processing stimuli from the surroundings and handling relations to other people. This can make the residents outward reacting or shut off from the surroundings. Activities should therefore be simple, passive, and staff led with a focus on experiencing nature sitting or lying down comfortably.

It is important to bear in mind that the Nature Dementia Support Model only sketches out a general strategy based on an overall approach to the needs of persons with dementia.

### 3.2 Practitioner use of the nature dementia support model

By presenting the architectural proposal for Tune nursing home, the following illustrates how the Nature Dementia Support Model can be used in practice to facilitate architectural programming and the design of green nursing home environments intended to be supportive.

#### 3.2.1 Overall description of the architectural proposal

**Figure 2:**
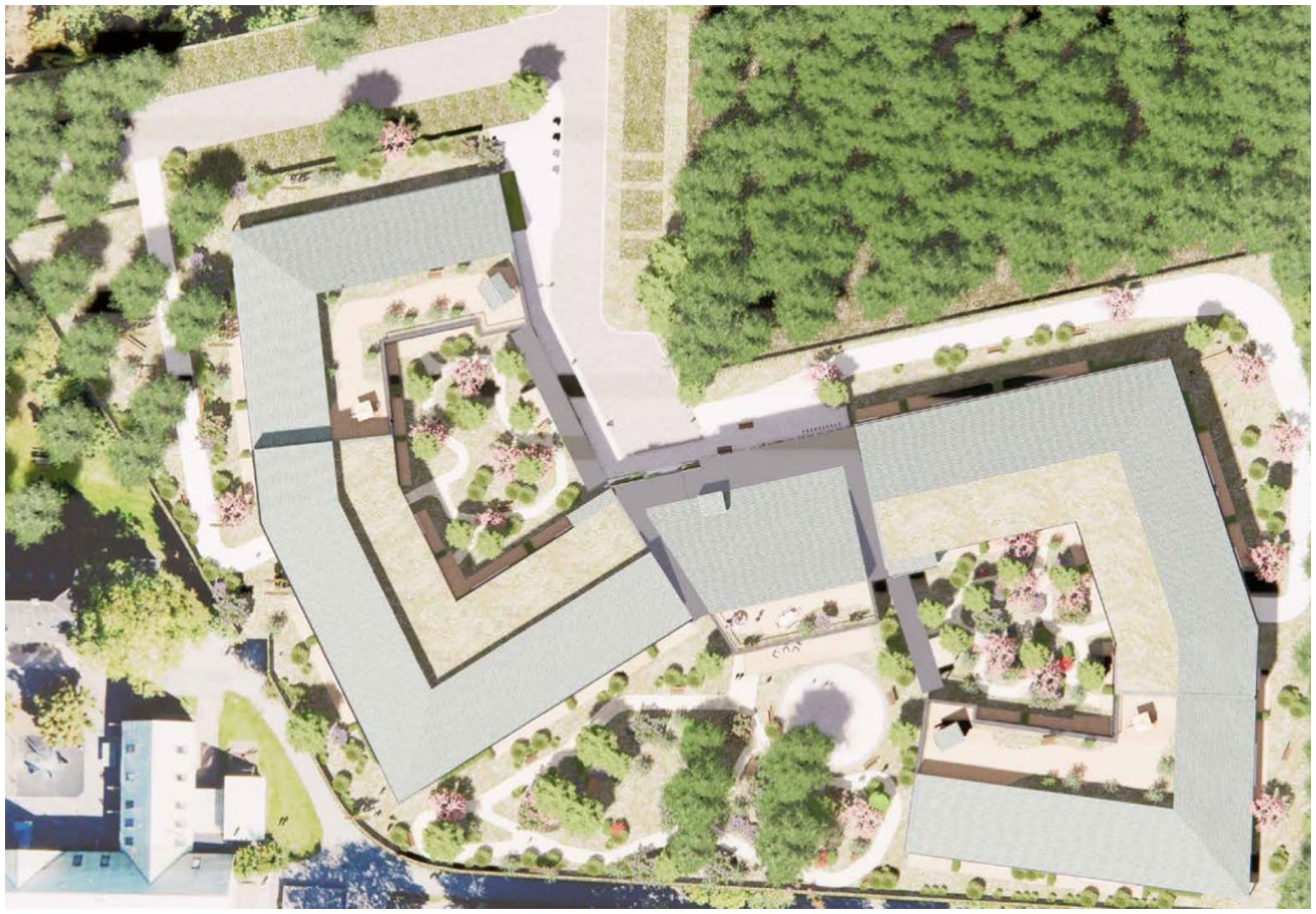
Site plan of tune nursing home and the nearest surroundings including a small urban woodland. Illustration credit: Vandkunsten Architects.

**Figure 3:**
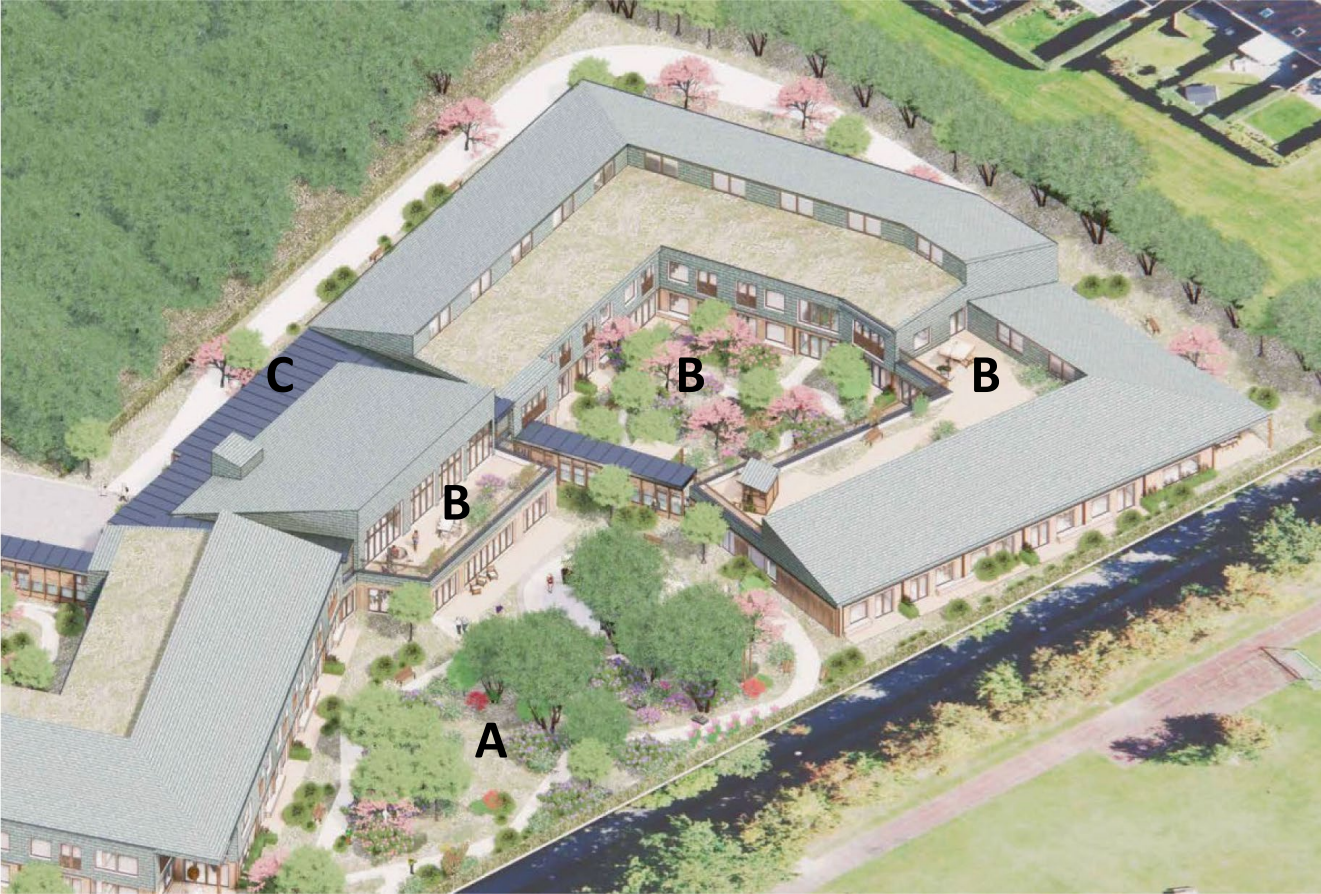
Axonometric projection of tune nursing home. The three types of green environments include; (A) a park, (B) courtyards and rooftop terraces, and (C) a green and light-filled indoor room. Illustration credit: Vandkunsten Architects.

**Figure 4:**
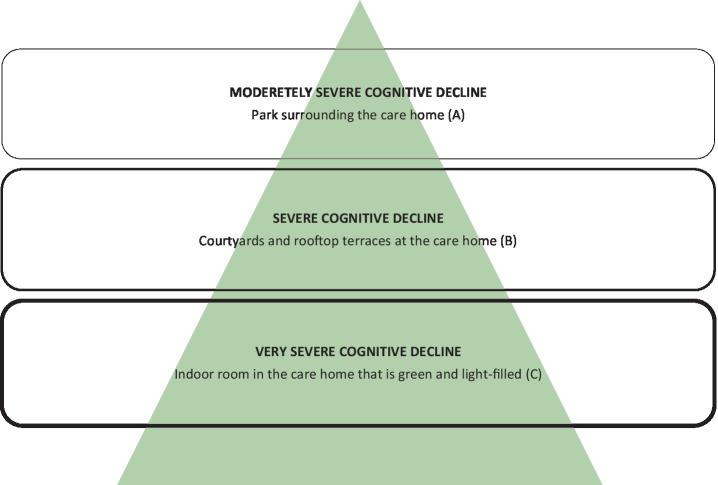
The Nature Support Model for Dementia illustrating the stages of decline from top to bottom with their corresponding type of green environment at Tune nursing home.

The visionary strategy to use green environments and nature to support resident well-being throughout the progression of dementia, was decisive in the design of Tune nursing home. The size of the building and the site as well as the location of the nursing home (which is connected to a small urban woodland), made this inclusion possible in different ways. In line with the Nature Support Model for Dementia, Vandkunsten Architects worked with a concept that includes three different types of green environments: (A) the park surrounding the nursing home; (B) courtyards and rooftop terraces at the nursing home; (C) an indoor room that is green and light-filled inside the nursing home (see Figures 2, 3). The three environments cater to people with moderately severe, severe, and very severe dementia, respectively (see Figure 4).

Each of these green environments are described below, with a focus on the way they meet the needs in the three final stages of dementia. At the outset, all areas were designed to follow the general design guidelines from the reviews listed above. In addition, they are differentiated in the degree to which they relate residents to non-human environments, scope of action, assistance as well as safety, and security (as conceptually illustrated in Figure 4).

#### 3.2.2 Park (A)

**Figure 5:**
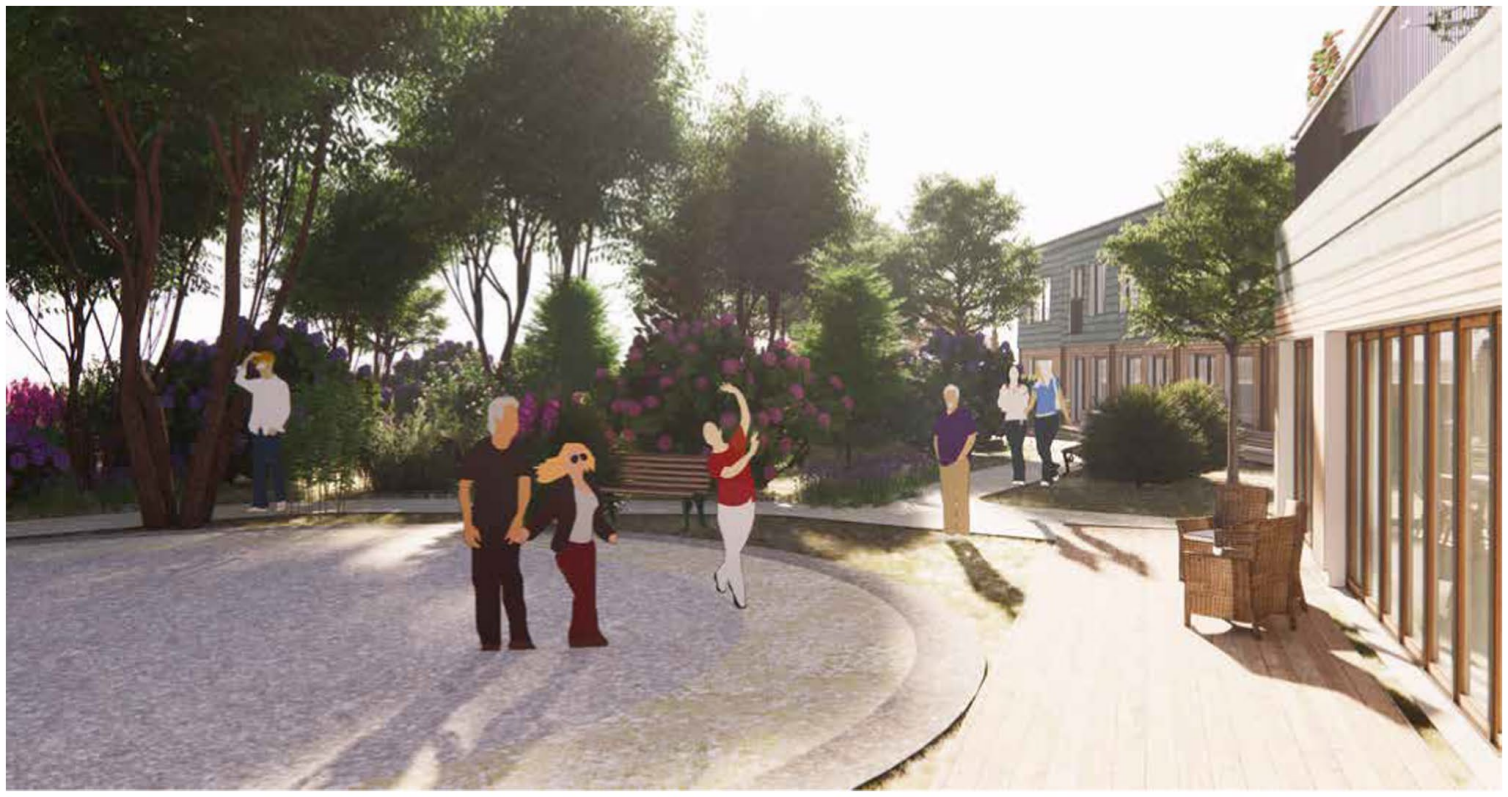
Park. Illustration credit: Vandkunsten Architects.

The park of approx. 1000m^2^ is a green outdoor environment surrounding Tune nursing home catering to people with moderate dementia. The boundaries are defined by a wooden country-style fence which at certain points provides gated access to the surroundings which includes a small urban woodland. The park consists of a lightly undulating meadow with scattered bushes and trees and a winding strolling path inviting curiosity and access to the different areas of the park. The grounds include a vegetable garden affording meaningful activities and reminiscence, a rose garden which stimulates the senses, and an area with ergonomic exercising equipment. The park also features a covered swing for a tête-à-tête, and a dance stage for socializing and exercising in groups (see Figure 5). In the park, active participation is generally emphasized, providing areas for activity, exercise, and socialization.

#### 3.2.3 Courtyards and rooftop terraces (B)

**Figure 6:**
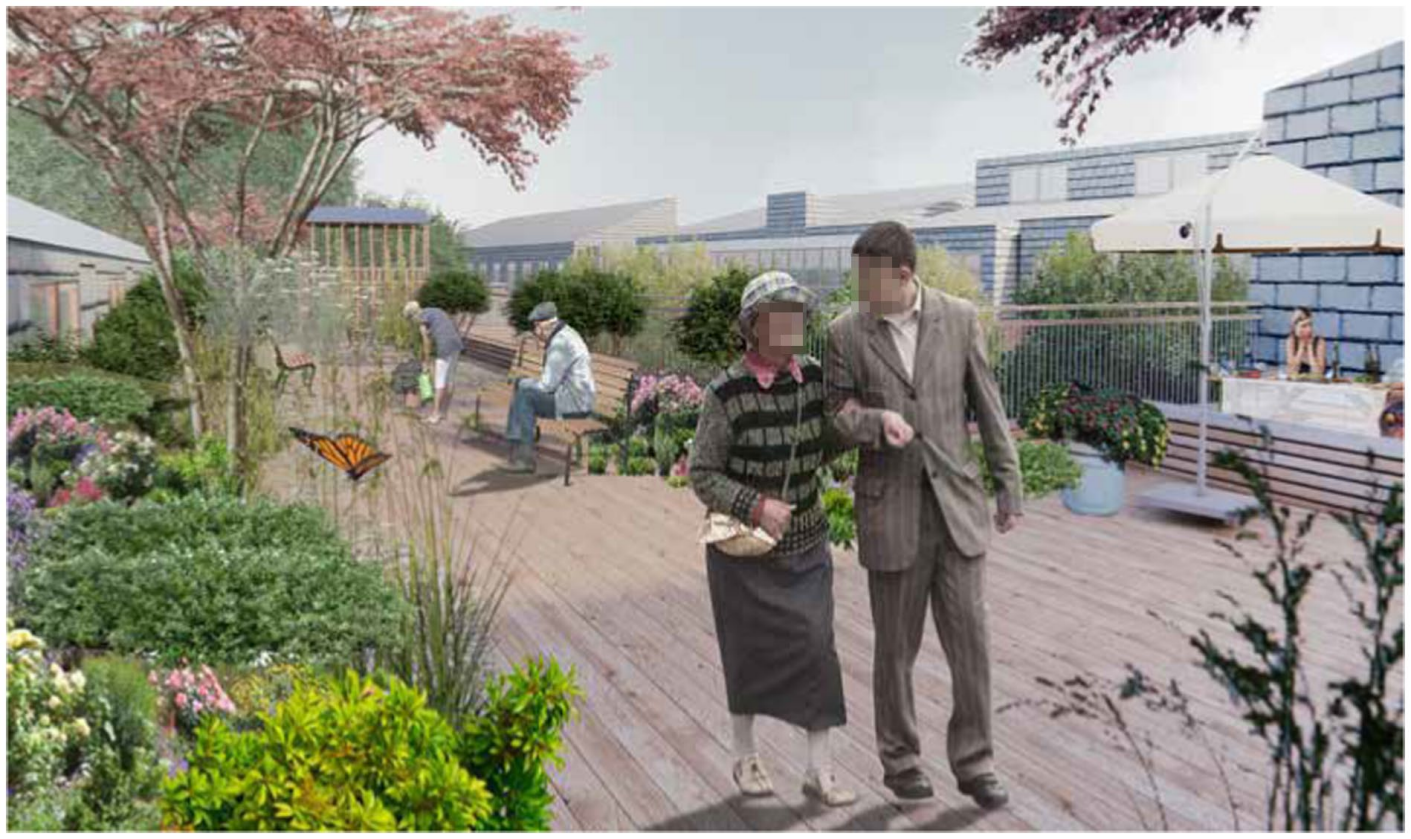
Rooftop terraces. Illustration credit: Vandkunsten Architects.

The courtyards and rooftop terraces are green outdoor environments that are securely enclosed by the building. They are intended for people with severe dementia and have proximity and easy access to indoor facilities such as resting areas and restrooms as well as staff assistance, all adding to a sense of safety. Both the courtyards and rooftop terrasses are green and provide access to trees, bushes, and flowering perennials via a path or a deck (see Figure 6). Still, their limited size (approx. 50 – 250m^2^) make them easy to orientate oneself within. Different types of seating offer rest areas for small groups or solitary sitting. A teahouse, a pavilion, and parasols provide sheltered seating options. These are both places for activity (such as participating in a tea party) and retreat (e.g., in one of the two outdoor bathtubs).

#### 3.2.4 Green and light-filled indoor room (C)

**Figure 7:**
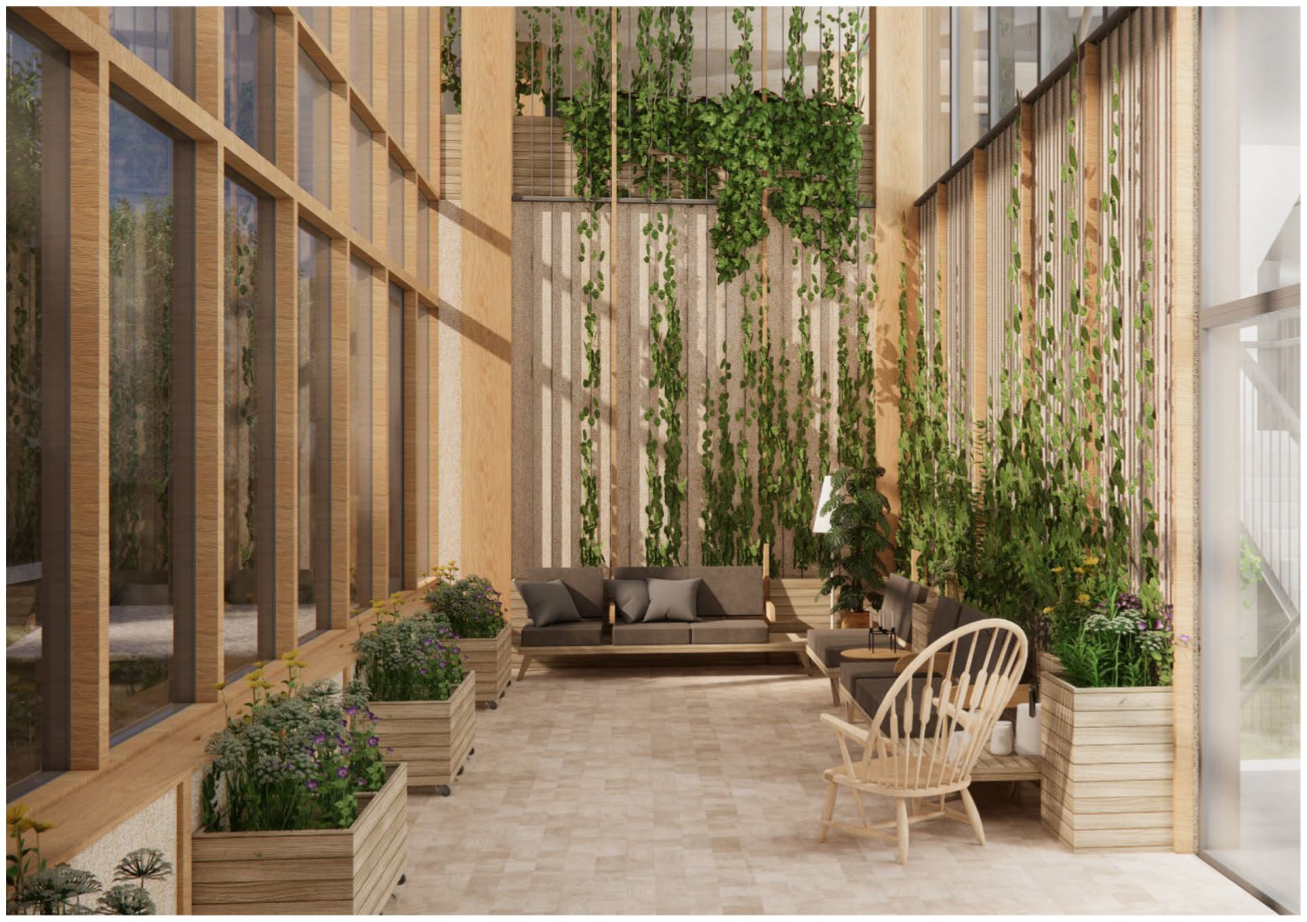
Green and light-filled indoor room. Illustration credit: Vandkunsten Architects.

The green and light-filled indoor room is quite small (approx. 50m^2^), is closed off from the rest of the nursing home, and is located with views to the surrounding park and urban woodland. It is intended for people with very severe dementia and caters a high level of staff assistance as well as passive and simple activities such as looking at the view and enjoying the green atmosphere in the room. It’s a safe prospect-refuge retreat providing contact with nature indoors. It features comfortable resting areas where one can sit or lie down. Sensory stimuli such as nature views, light, sounds, tactility, and smells are adjusted to people with high sensitivity aiming to provide a pleasant and comforting experience. Plants are non-toxic. The room faces north which protects it from direct sunlight. At the same time, it is high-ceilinged and has large windows which gives it good exposure to the light of the sky (see Figure 7). Curtains enable occupants to adjust light exposure for the specific user. Indoor trees, scented plants, and soft materials such as textiles, upholstery, and polished wood add to the soothing atmosphere. Floor heating and airflow keep room temperature and odor nuisances in check. Special care is also taken to address acoustics using sound absorbing materials. Finally, a water feature adds the soothing sounds of gurling water.

## 4 Discussion

When separate green nursing home environments are adjusted to cater to the three last stages of dementia respectively, instead of providing only one common green environment, this results in several advantages. First, it is possible to adjust the environments specifically to the needs of residents, which vary significantly between the three last stages of dementia progression. This optimizes the health benefits of contact with nature in nursing homes. As an example, having a specific outdoor environment for each of the stages can potentially lead to more interaction for those who seek more complex relations (e.g., with other residents) and more peace for those who seek a simple relation to nature. Secondly, in terms of care, the uniformity among users of a given green environment can make it easier for staff to carry out work and plan for resident needs. If a nursing home only has space for one green environment, it may be possible to work with a graduation from one type of area to the next in the same environment, depending on the scale for the green space. This could be an indoor winter garden that leads to a terrace, around which there is a garden. It is important to work with transitions between the areas in a way that caters for overview and accessibility as well as safety and security at the same time.

In Figure 1 a discrepancy can be seen in that people in the last stage of dementia have the highest need for assistance from staff and at the same time the highest need for a non-human environment. People in this stage are completely dependent on help from staff. These residents tend to become overwhelmed by sensory impressions and can have a hard time interacting with other people due to intellectual and emotional impairment. Here lies a challenge in finding the balance between information overload and lack of assistance. Having a simple and controllable green environment that staff can adjust to each individual can be essential to finding this balance.

An extensive study focusing on uncovering which factors had an influence on the use of green environments at nursing homes showed that the commitment of management and staff was of great importance (Grant and Wineman, 2007). It is crucial that there is a clear-cut purpose for how a nursing home wants to use nature as a part of care and that the staff and management commit to this approach. This could take the form of a detailed plan of action describing the use of each green environment and the health goals related to it. Opting for sustainable planting and prioritizing maintenance of the green environments are also important parts of this commitment.

## 5 Conclusion

Including green environments in nursing homes so that people with dementia may be in contact with nature can hold many benefits if done in a purposeful way. First, it can support resident well-being. Second, it can lower agitation and improve the affect and attention of residents, which can reduce the demands on staff and improve their working conditions. Thirdly, having a nice place to stay and meaningful things to do during visits can encourage friends and family to spend time with their relatives. Hopefully the Nature Support Model for Dementia can help structure the programming and design of supportive nursing home environments. An important task ahead is to test the model in a post occupancy evaluation and improve it according to the results, creating new evidence for designing with the overall goal being to assist in taking good care of the elderly and facilitating decline with dignity.

## Data Availability

The original contributions presented in the study are included in the article, further inquiries can be directed to the corresponding author.
